# 3D in vitro M2 macrophage model to mimic modulation of tissue repair

**DOI:** 10.1038/s41536-021-00193-5

**Published:** 2021-11-30

**Authors:** Jiranuwat Sapudom, Shaza Karaman, Walaa K. E. Mohamed, Anna Garcia-Sabaté, Brian C. Quartey, Jeremy C. M. Teo

**Affiliations:** 1 Laboratory for Immuno Bioengineering Research and Applications, Division of Engineering, New York University Abu Dhabi, Abu Dhabi, UAE; 2 Department of Biomedical Engineering, Imperial College London, London, UK; 3 Department of Mechanical and Biomedical Engineering, Tandon School of Engineering, New York University, New York, NY USA

**Keywords:** Regenerative medicine, Tissue engineering, Immunology

## Abstract

Distinct anti-inflammatory macrophage (M2) subtypes, namely M2a and M2c, are reported to modulate the tissue repair process tightly and chronologically by modulating fibroblast differentiation state and functions. To establish a well-defined three-dimensional (3D) cell culture model to mimic the tissue repair process, we utilized THP-1 human monocytic cells and a 3D collagen matrix as a biomimetic tissue model. THP-1 cells were differentiated into macrophages, and activated using IL-4/IL-13 (M_IL-4/IL-13_) and IL-10 (M_IL-10_). Both activated macrophages were characterized by both their cell surface marker expression and cytokine secretion profile. Our cell characterization suggested that M_IL-4/IL-13_ and M_IL-10_ demonstrate M2a- and M2c-like subtypes, respectively. To mimic the initial and resolution phases during the tissue repair, both activated macrophages were co-cultured with fibroblasts and myofibroblasts. We showed that M_IL-4/IL-13_ were able to promote matrix synthesis and remodeling by induction of myofibroblast differentiation via transforming growth factor beta-1 (TGF-β1). On the contrary, M_IL-10_ demonstrated the ability to resolve the tissue repair process by dedifferentiation of myofibroblast via IL-10 secretion. Overall, our study demonstrated the importance and the exact roles of M2a and M2c-like macrophage subtypes in coordinating tissue repair in a biomimetic model. The established model can be applied for high-throughput platforms for improving tissue healing and anti-fibrotic drugs testing, as well as other biomedical studies.

## Introduction

Wound healing is a complex and dynamic process facilitated by four overlapping, continuous phases namely homeostasis, inflammation, proliferation, and tissue remodeling^1,2^. This entire process is tightly modulated and orchestrated by biochemical signals, such as growth factors and cytokines, and biophysical stimuli including cell–cell interactions and cell–extracellular matrix (ECM) interactions^3^. Various cell types, including immune cells, fibroblasts, endothelial cells, and keratinocytes are intricately involved in the wound-healing process^4,5^. In the early phase of wound repair, fibroblasts proliferate and migrate from adjacent tissues towards the wound bed via chemotaxis, in order to form granulation tissue and synthesize new ECM^6^. Fibronectin is a high-molecular weight adhesive glycoprotein that originates from blood plasma and becomes part of the wound matrix due to the rupture of localized vasculature during tissue injury. Fibronectin has also been reported to enhance migration of fibroblasts into wound tissue and stimulate their proliferation^7,8^. Afterwards, fibroblasts differentiate into myofibroblasts via transforming growth factor beta-1 (TGF-β1)-mediated Smad2/3 signaling^9,10^. Recent reports suggest that attenuation of TGF-β1 signaling through the use of glycosaminoglycan-based hydrogel reduce scar formation in rat dermal wounds^11,12^, while excessive reduction of TGF-β1 can be paradoxical, delaying wound repair^13^. Myofibroblasts are characterized by pronounced actin stress fibers and the expression of alpha smooth-muscle actin (αSMA), as well as an extensive production and deposition of ECM proteins, e.g. type I collagen (Coll I) and fibronectin containing extra domain-A (EDA-FN)^8,14,15^. In addition, they are able to reorganize collagen fibrils of the ECM using their contractile machinery, actively playing a role in physically closing the wound^16,17^. Alongside with that, collagen fibril density is increased during the tissue repair process and, in turn, can act as a negative feedback to downregulate myofibroblastic activity^18,19^. However, if the tissue repair is prolonged and myofibroblasts remain to produce excessive ECM components and steady remodel the tissue, it will result in scarring or fibrosis^20,21^.

Macrophages are known as the most dominating cells at the wound site which coordinate the transition between phases during the entire wound-healing process^22–24^. In particular, anti-inflammatory macrophages, known as M2 macrophages, are key regulators in the initiation and resolution of tissue repair phase by secreting specific cytokines to modulate fibroblast fates^25,26^. In classical macrophage biology, M2 macrophages are classified into distinct subtypes, namely M2a, M2b, M2c, M2d, and M2f based on their marker expressions and cytokine secretion profiles^27^. Macrophage phenotype is in fact a continuous spectrum; however, description through their distinct subtypes provides clarity^28^. It has been hypothesized that the balance between regenerative wound healing and scarring/fibrosis pivots on the equilibrium between the M2a and M2c phenotype, each with distinct cellular functions. However, few studies have investigated the role of M2a and M2c in other physiological contexts^29–31^ and even less is known about how both cell subtypes regulate fibroblast differentiation and functions.

The limitation of the current in vitro wound-healing model is that most of the previous models were conducted on two-dimensional (2D) tissue culture plastic, which lacked representative ECM components and exhibited non-physiological features, such as high mechanical stiffness and lack of porosity^32–34^. To bridge the gap between 2D cell culture models and native tissues, three-dimensional (3D) fibrillar collagen matrices can be utilized to mimic wound tissue, since it recapitulates the fibrillar microstructure of native tissues^18,35,36^. In addition, the 3D collagen systems could be fine-tuned in terms of their biophysical properties, e.g., pore size, fibril size and elasticity^33^, as well as model tissue compartments^37^. Besides, they can be post-modified with other ECM proteins such as fibronectin and glycosaminoglycans to enhance the complexity of the biomimetic models^33^.

The aim of this work is to establish THP-1-derived M2 subtypes, namely M2a and M2c, for utilization in a well-defined 3D collagen-based wound-healing model, facilitating the development of high-throughput platforms for models of wound healing. First, phenotypes of both macrophage subtypes were comprehensively characterized in terms of expression of cell surface markers and cytokine secretion profiles. Afterwards, fibroblasts and myofibroblasts were each co-cultured with both M2 subtypes and studied in terms of their differentiation, matrix protein production, matrix remodeling, and migratory behavior.

## Results and discussion

### Establish 3D in vitro model and macrophage morphology

Current wound-healing models are based on 2D tissue culture plastic, lacking many biophysical and biochemical features found in native wound tissue. In this work, we aimed to establish a biomimetic 3D cell culture models to replicate modulation of the initiation and resolution phases of the tissue repair. Our cell culture model facilitates the development of well-defined wound-healing models for high-throughput screening in drug development, studying of cell–cell and cell–matrix interactions, as well as other biomedical studies. We utilized 3D collagen matrices as a 3D cell culture model. Our 3D collagen matrices reconstituted at 2 mg/ml collagen concentration, exhibit a pore size of 8.1 ± 0.8 µm, a fibril diameter of 652.0 ± 32.0 nm, and an elastic modulus of 85.5 ± 7.3 Pa.

**Fig. 1:**
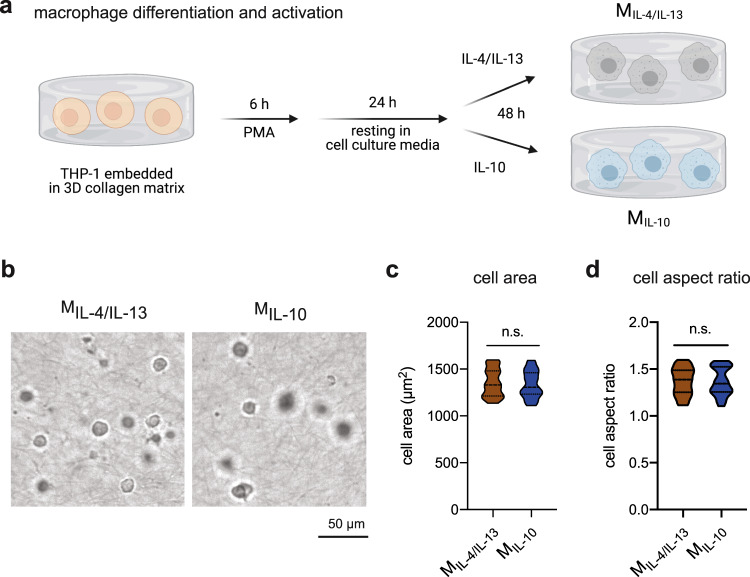
Macrophage differentiation and activation in 3D collagen matrices. **a** Schematic workflow for macrophage differentiation and activation towards M_IL-4/IL-13_ and M_IL-10_ in 3D collagen matrix. **b** Representative images of M_IL-4/IL-13_ and M_IL-10_ were gathered using bright-field microscopy. A quantitative morphological analysis, namely **c** cell area and **d** cell aspect ratio, was conducted using an image analysis toolbox. Data are presented as a violin plot. At least 100 cells from three independent experiments were analyzed.

As high-throughput studies require large number of cells and to overcome donor-to-donor variation of primary cells, we utilized THP-1-derived macrophages as a cell model. To generate M2 macrophage subtypes, we first differentiated embedded THP-1 cells into uncommitted macrophages using PMA and further activated them using interleukin-4 (IL-4)/IL-13 and IL-10 into M_IL-4/IL-13_ and M_IL-10_, respectively (Fig. 1a). After macrophage activation, we quantified cell morphology by means of measuring cell area (Fig. 1c) and cell aspect ratio (Fig. 1d) using an automated image analysis toolbox^38^. Both M_IL-4/IL-13_ and M_IL-10_ cells exhibited a round morphological appearance, as visualized in Fig. 1b and quantified by cell aspect ratio (Fig. 1d), no significant difference could be found between both macrophage subtypes. Macrophages are distributed homogeneously throughout 3D collagen matrices (Supplementary Fig. 1). For further characterization, we comprehensively studied cell surface markers and cytokine secretion profile. Later, both activated macrophages subtypes were co-cultured with fibroblasts and myofibroblasts to demonstrate differential cellular functions. Specifically, we aim to demonstrate the advantage of our 3D cell culture models for in-depth study of collagen reorganization during the initiation and resolution phases of the tissue repair.

### Establish and characterization of M_IL-4/IL-13_ and M_IL-10_ macrophage

**Fig. 2:**
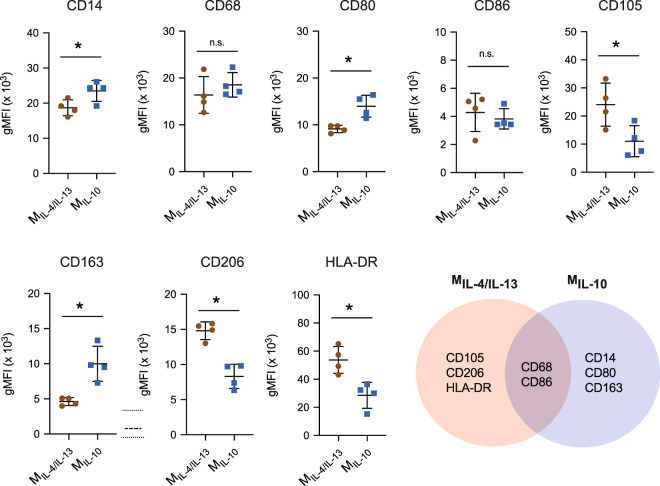
Quantitative analysis of cell surface markers of M_IL-4/IL-13_ and M_IL-10_. Cell surface markers were analyzed by immunocytostaining of CD14, CD68, CD80, CD86, CD105, CD163, CD206, and HLA-DR. Geometric mean fluorescence intensity (gMFI) was plotted. Black line and error bar in the plot represent mean and standard deviation, respectively. * indicates a significance level of *p* ≤ 0.05 using Mann–Whitney test. Experiments were performed in four independent replicates.

To distinguish between the macrophage subtypes used in this study, we first characterized both M_IL-4/IL-13_ and M_IL-10_ cells using their surface marker profile. The expression of CD14, CD68, CD80, CD86, CD105, CD163, CD206, and HLA-DR was measured via immunocytostaining using flow cytometry and quantified by geometric mean fluorescence intensity (gMFI) (Fig. 2). Histogram plots of quantified surface markers are depicted in Supplementary Fig. 2.

As shown in Fig. 2, both M2 subtypes shared similar expression levels of CD68 and CD86. Since CD68 is a pan-macrophage marker^39^, it is not surprising that a recent report suggested expression level of CD68 increased upon differentiation in THP-1-derived macrophages, as compared to undifferentiated cell^40^. We further showed that THP-1-derived macrophages activated towards pro-inflammatory subtype and treated using IL-4/IL-13 showed no difference in CD68 expression^40^. CD86, on the other hand, is reported to be highly expressed in pro-inflammatory macrophages^39^ and hence used to discriminate M2a and M2c from pro-inflammatory macrophages^41^.

M_IL-4/IL-13_ exhibited higher expression levels of CD105, CD206, and HLA-DR when compared to M_IL-10_. HLA-DR is generally expressed approximately 5- to 10-fold higher in pro-inflammatory macrophages when compared to anti-inflammatory macrophages for both PBMC- and THP-1-derived macrophages^27,42^. In this work, we found a higher expression of HLA-DR in M_IL-4/IL-13_ when compared to M_IL-10_ macrophages, suggesting a higher pro-inflammatory activity of M_IL-4/IL-13._ As for the expression of CD105 and CD206, it is reported that both these surface markers can be induced by IL-4/IL-13 in both PBMC- and THP-1-derived macrophages^24,25^, explaining the high expression of CD105 and CD206 measured in our M_IL-4/IL-13_. We previously showed that THP-1-derived pro-inflammatory macrophages and treated with IL-4/IL-13 showed similar levels of CD206.

M_IL-10_ expressed high CD14, CD80, and CD163. CD14 is known used as an M2c-specific marker^31^. It has been showed that IL-10 enhanced CD14 expression^43^. In general, CD80 surface markers have been reported to be highly expressed in pro-inflammatory macrophages^44,45^. However, a recent report demonstrated that the presence of CD80 in anti-inflammatory macrophages is involved in the differentiation of regulatory T cells which play an important role in anti-rejection of transplants and maintaining tolerance^46,47^. CD163 is identified as an anti-inflammatory receptor, which expression can be induced by IL-10 (ref. ^48^). The high expression of CD14, CD80, and CD163 suggest an advanced suppressive role of pro-inflammatory response^49,50^.

**Fig. 3:**
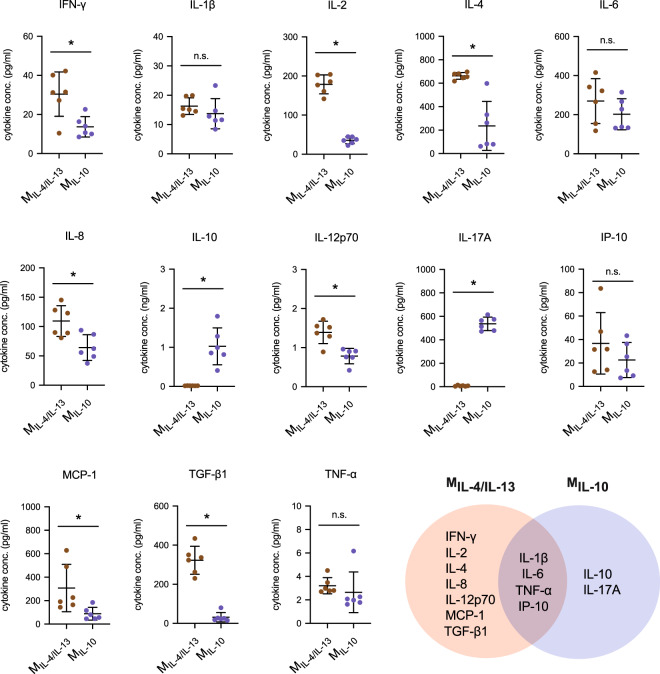
Quantitative analysis of cytokine secretion profile of M_IL-4/IL-13_ and M_IL-10_. Cytokine secretion profile was analyzed from cell culture supernatants after 3 days of macrophage activation by a bead-based multiplex immunoassay using flow cytometry. Data are shown as a dot plot. Black line and error bar in the plot represent mean and standard deviation, respectively. * indicates a significance level of *p* ≤ 0.05 using Mann–Whitney test. Experiments were performed in six independent replicates.

To further characterize both M2 phenotypes, we studied the cytokine secretion profile using a multiplex bead-based immunoassay (Fig. 3). To ensure that IL-4, IL-10, and IL-13 were autologously secreted, and not absorbed and released by the collagen through our exogenous application during macrophage activation, collagen matrices were incubated with those cytokines and the release profile in the media was determined over 3 days. Additionally, matrices were digested using collagenase and cytokine levels were quantified. Both soluble or matrix-bound IL-4, IL-10, and IL-13 were undetectable using the bead-based immunoassay, and therefore quantified cytokines were secreted by cells.

As shown in Fig. 3, we found significantly higher secretion levels of interferon-γ (IFN-γ), IL-2, IL-4, IL-8, IL-12p70, MCP-1, and TGF-β1 in M_IL-4/IL-13_ when compared to M_IL-10_. Both cells secreted similar levels of IL-1β, IL-6, tumor necrosis factor-ɑ (TNF-ɑ), and IP-10. In general, IFN-γ, IL-1β, IL-6, IL-12p70, IP-10, and TNF-ɑ are pro-inflammatory cytokines secreted by macrophages during the early wound-healing process^51^; however, they are generally expressed by M1 macrophages^18,40,52^. Especially, IFN-γ, IL-2, IL-4, and IL-12 have been associated with accelerated tissue repair^53,54^ and reported to contribute to effective wound healing. Any dysregulation in the secretion of those cytokines can lead to tissue fibrosis^55–58^. In addition to pro-inflammatory cytokines, M_IL-4/IL-13_ secreted a high amount of TGF-β1, a well-known multifunctional cytokine involved in the process of fibroblast differentiation. It has been demonstrated that TGF-β1 is secreted by macrophages activated with IL-4/IL-13 in both PBMC and THP-1-derived macrophages^18,30^. The secretion of TGF-β1 also supports the maintenance of M2a phenotype^59^.

On the other hand, M_IL-10_ produced high amounts of IL-17 and IL-10 which were undetectable from M_IL-4/IL-13_. While most analyzed pro-inflammatory cytokines have been demonstrated to enhance tissue repair, IL-17 is a pro-inflammatory cytokine that is involved in the activation of M2 macrophages via NFκB signaling^60^ and the administration of recombinant IL-17A leads to delayed wound healing^61^, which might relate to stimulation of differentiation of human anti-inflammatory macrophages M2c in response to IL-10 (ref. ^62^). IL-10 is the anti-inflammatory cytokine reported to be highly secreted in IL-10 activated PBMC-derived macrophages (M2c) in particular^52^. It is coupled with the regulation of the different phases during wound healing^35,63–66^.

The cytokine secretion profile of our M_IL-4/IL-13_ and M_IL-10_ matches that of PBMC-derived M2a and M2c, respectively^52^. However, it will be incorrect to compare actual levels of secreted cytokines from both PBMC- and THP-1-derived macrophages due to experimental differences in cell number, activation protocols, cell culture dimensionality, biophysical and biochemical properties of cell culture substrate, and cell culture conditions. It should be reiterated that our cytokine analysis is based on activated macrophages in 3D cell culture models. We previously have reported that many cytokines, including IL-1, IL-6, IL-8, IL-10, IL-12, MCP-1, and TGF-β1, are secreted in collagen density-dependent manner by macrophages^18^. In that latter study, pro-inflammatory cytokines, IL-1β, IL-6, IL-8, and TNFα, were elevated in macrophages treated with LPS/IFNγ, namely pro-inflammatory macrophages as compared to M_IL-4/IL-13_.

In sum, surface markers and cytokine secretion profiles suggest that M_IL-4/IL-13_ and M_IL-10_ activated macrophages demonstrate distinct M2 phenotypes, which are in line with previous findings on M2a and M2c macrophage subtypes in primary human macrophages^22,28,52^, respectively.

### Mimicking the early phase of tissue repair

**Fig. 4:**
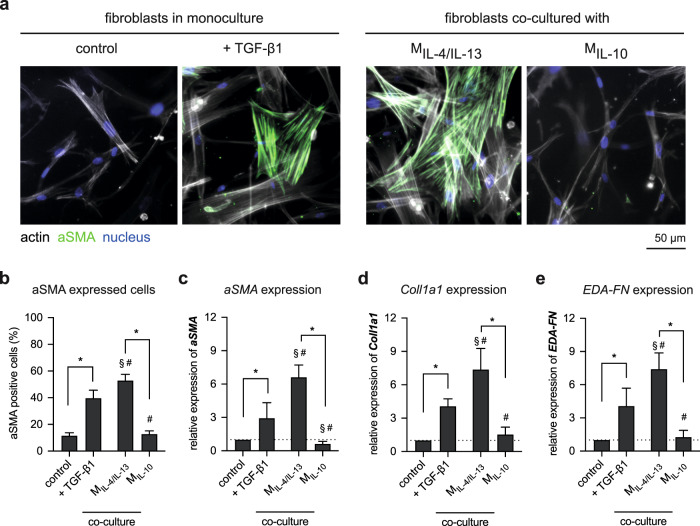
Alteration of fibroblast morphology and specific markers in the presence of TGF-β1 and co-culture with M2 macrophages subtypes. **a** Representative images showing nuclei (blue), actin filaments (gray), and αSMA (green) (scale bar: 50 μm). Fibroblast differentiation was analyzed by **b** manual counting of αSMA-positive cells. At least 200 cells per condition were counted. Gene expression analysis of **c** *αSMA*, **d** *Coll1a1*, and **e** *EDA-FN* were performed using qPCR. Data are represented as mean ± SD; *a significance level of *p* ≤ 0.05 using a one-way ANOVA followed by Tukey’s post hoc analysis. The characters # and § represent the significance level of *p* ≤ 0.05 using a one-way ANOVA followed by Tukey’s post hoc analysis when compared to control, and treated samples with TGF-β1, respectively. Experiments were performed in four replicates.

To further differentiate between both M2 phenotypes and demonstrate their potential in regulating fibroblast differentiation during the early phase of tissue repair, human primary dermal fibroblasts were co-cultured with M2 phenotypes for 3 days and myofibroblast-related genes were analyzed using qPCR analysis (Fig. 4). Respectively, as negative and positive control of myofibroblast differentiation, fibroblasts were monocultured in the absence and presence of 10 ng/ml TGF-β1 for 3 days. TGF-β1 is known to trigger fibroblast differentiation in vitro^21,67^. Fibroblasts cultured in the presence of TGF-β1 and co-cultured with our M_IL-4/IL-13_ exhibited more pronounced actin stress fibers with αSMA incorporation as well as a more flattened cell morphology, suggesting a differentiation of fibroblast into myofibroblast (Fig. 4a). This could not be observed in untreated fibroblasts (control) and those co-cultured with M_IL-10_.

To confirm the fibroblast differentiation, we quantified αSMA expression, a myofibroblast marker^8,68^, using immunocytostaining (Fig. 4b) and gene expression (Fig. 4c) analysis. αSMA-positive cells and gene expression increased in fibroblast culture treated with TGF-β1 and when co-cultured with M_IL-4/IL-13_, when compared to control and co-cultured with M_IL-10_. Both αSMA-positive cells and gene expression correlated nicely. This result also corroborates with previous reports which demonstrate the effect of TGF-β1 (refs. ^8,66,69^) and M_IL-4/IL-13_ mediated fibroblast differentiation^18^. Besides αSMA expression, we analyzed matrix protein production potential, through analyzing alpha 1 chain of the type 1 collagen (Coll1a1) and fibronectin containing Extra Domain-A (EDA-FN). The expression of both *Coll1a1* (Fig. 4d) and *EDA-FN* (Fig. 4e) follow the same trend of αSMA expression. Again, *Coll1a1* and *EDA-FN* were highly expressed in fibroblast co-cultured with M_IL-4/IL-13_ rather than TGF-β1-treated fibroblasts. Fibroblasts co-cultured with M_IL-10_ did not show an enhanced expression and the expression level of both matrix proteins was in the same range as untreated fibroblasts.

In sum, by co-culturing fibroblasts with both macrophage subtypes, we found that only M_IL-4/IL-13_ is able to differentiate fibroblast into myofibroblast, as demonstrated by αSMA and matrix protein expression. This could be explained by the higher TGF-β1 expression in M_IL-4/IL-13_ when compared to M_IL-10_ (Fig. 3). Interestingly, the expression levels of αSMA and matrix protein in fibroblast co-cultured with M_IL-4/IL-13_ are significantly higher than fibroblast directly treated with TGF-β1, which is to our previous finding^18^. This variation could be explained by the sustained release of TGF-β1 by M_IL-4/IL-13_. As demonstrated using controlled release microbeads, pulsed paracrine delivery of TGF-β1 in a picogram range (100 pg/ml) from polyethylene glycol (PEG) microbeads embedded in 3D collagen matrix could fully differentiate fibroblasts into myofibroblast similar to a continuous 10 ng/ml systematic delivery^69^.

**Fig. 5:**
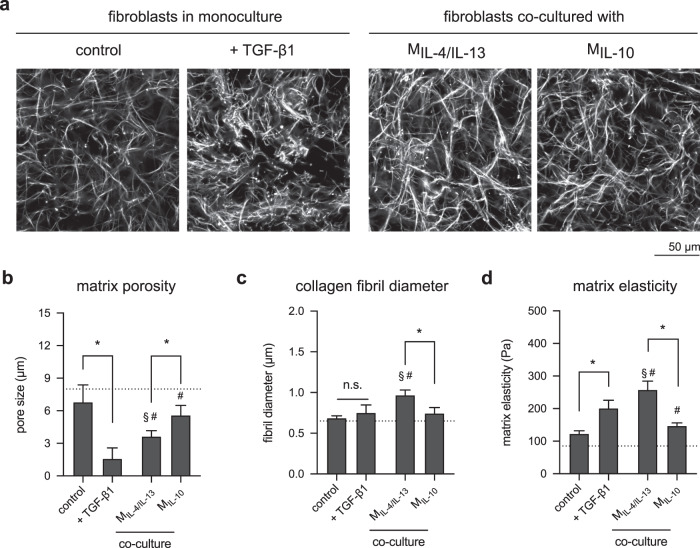
Matrix remodeling by fibroblasts in the presence of TGF-β1 and co-culture with M2 subtypes. **a** Representative images of decellularized collagen matrices in mono and co-culture with M2 subtypes (scale bar: 50 μm). Decellularized collagen matrices were characterized regarding **b** matrix porosity characterized as mean pore size, **c** collagen fibril diameter, and **d** bulk matrix elastic modulus (data are represented as mean ± SD; *significance level of *p* ≤ 0.05 using a one-way ANOVA followed by Tukey’s post hoc analysis). The characters # and § represent the significance level of *p* ≤ 0.05 using a one-way ANOVA followed by Tukey’s post hoc analysis when compared to control, and treated samples with TGF-β1, respectively. For the quantification of matrix porosity and collagen fibril diameter, 10 different positions of each matrix condition were analyzed. Experiments were performed in four replicates.

Besides production of matrix proteins, another major feature reserved for myofibroblasts is their ability to remodel the matrix. Collagen matrices were decellularized using osmotic pressure by incubating in bi-distilled water and stained using TAMRA-SE for visualization by confocal laser microscopy (Fig. 5a). Changes in matrix characteristics could be visually observed. The quantitative analysis of matrix porosity and fibril diameter using a custom-built image analysis toolbox demonstrated that matrices of TGF-β1 treated fibroblasts exhibit a higher collagen density than the untreated counterpart and matrices of fibroblast co-cultured with both M2 macrophage (Fig. 5b). Matrices of fibroblast co-cultured with M_IL-4/IL-13_ showed significantly smaller pore size than when co-cultured with M_IL-10_. Interestingly, an increase in collagen fibril diameter was only found in matrices of fibroblast co-cultured with M_IL-4/IL-13_. This observation is consistent with the initial development of fibrosis, where an increase of collagen fibril can be found in pro-inflammatory milieu^70^, which at low levels, our M_IL-4/IL-13_ are able to secrete (Fig. 3). In addition, it has been reported that collagen fibril diameter can mechanically actuate myofibroblast differentiation^71^, which might also explain why fibroblast differentiation in co-culture with M_IL-4/IL-13_ exhibited higher expression of αSMA (Fig. 4b, c) and matrix proteins (Fig. 4d, e).

Furthermore, we analyzed the elastic modulus of the remodeled matrices using a contactless rheometer (Fig. 5d). Matrices of untreated fibroblast and fibroblast co-culture with M_IL-10_ showed a similar range of elastic modulus. Interestingly, matrices of fibroblast co-cultured with M_IL-4/IL-13_ showed higher elastic modulus than that from TGF-β1-treated fibroblasts. As demonstrated in various works^71–74^, the contribution of collagen fibril diameter on bulk matrix modulus is not negligible. For example, collagen matrices with bigger pore size and thicker fibrils demonstrated higher elastic modulus than matrices with smaller pore size and thinner fibrils^72,74^. These previous findings provide an explanation for our obtained results of mechanical analysis.

### Mimicking the resolution phase of tissue repair

While M_IL-4/IL-13_ has shown their potential in initiating tissue repair, M_IL-10_ has been hypothesized to terminate the tissue repair phase due to their low pro-inflammatory cytokine secretion and high secretion of IL-10 (Fig. 3). It has been reported that exogenously applied IL-10 is able to convert myofibroblasts into fibroblast^35,66^. However, few studies have been performed in co-culture conditions to investigate this phenomenon^35^. To address this, we first derived myofibroblasts by treating fibroblasts with 10 ng/ml TGF-β1 on 2D cell culture plastic for 3 days. Afterwards, myofibroblasts were detached and cultured on top of the collagen matrices pre-embedded with and without M2 subtypes. It has to be noted that the cell culture media of the myofibroblast in the control condition is TGF-β1 supplemented (10 ng/ml) to prevent myofibroblast apoptosis, as previously reported^66^. It is known that TGF-β1 can block the intrinsic apoptotic pathway by inhibiting the pro-apoptotic protein BCL-2-associated death promoter (BAD) via the FAK–PI3K–AKT signaling pathway in fibroblast^75^.

**Fig. 6:**
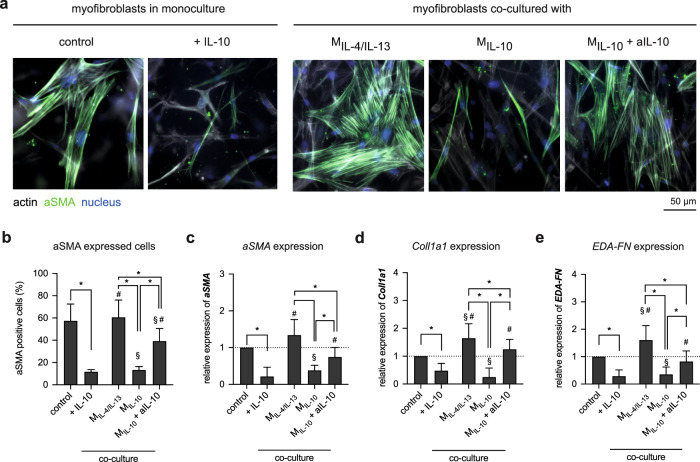
Alteration of myofibroblasts morphology and specific markers in the presence of IL-10 and co-culture with M2 macrophages subtypes. **a** Representative images showing nuclei (blue), actin filaments (gray), and αSMA (green) (scale bar: 50 μm). Myofibroblast de-dedifferentiation were analyzed using **b** percentage of αSMA-positive cells by manual counting of cells with αSMA incorporation into actin stress fibers and quantitative analysis of **c** *αSMA*, **d** *Coll1a1*, and **e** *EDA-FN* gene expression. For quantitative analysis of αSMA-positive cells, at least 200 cells per condition were counted. Data are represented as mean ± SD; *significance level of *p* ≤ 0.05 using a one-way ANOVA followed by Tukey’s post hoc analysis). The characters # and § represent the significance level of *p* ≤ 0.05 using a one-way ANOVA followed by Tukey’s post hoc analysis when compared to control, and treated samples with IL-10, respectively. Experiments were performed in four replicates.

While myofibroblasts in monoculture exhibit pronounced actin stress fibers with αSMA incorporation (Fig. 6a), this observation is attenuated when IL-10 is present systemically. Quantitative analysis of αSMA-positive cells (Fig. 6b) and α*SMA* expression (Fig. 6c) confirmed the observation, along with reduction matrix proteins expression, namely *Coll1a1* (Fig. 6d) and *EDA-FN* (Fig. 6e). In co-culture conditions, myofibroblastic phenotypes are maintained in the presence of M_IL-4/IL-13_, while myofibroblasts co-cultured with M_IL-10_ manifests a reduction in αSMA-positive cells and expression. The expression of matrix proteins was also reduced and correlated fittingly with the reduction of αSMA expression. Both results suggested the dedifferentiation of myofibroblast into fibroblast phenotypes when co-cultured with M_IL-10_, which is in line with dedifferentiation of myofibroblasts by PBMC-derived anti-inflammatory macrophages using macrophage colony-stimulating factor (M-CSF)^35^. To elucidate that myofibroblast dedifferentiation is related to the secretion of IL-10, we subsequently supplemented cell culture media with anti-IL-10 antibody (aIL-10). Results suggested that in the presence of aIL-10, the number of αSMA-positive cells and expression, as well as the expression of matrix proteins, remained at a similar level to control and co-culture with M_IL-4/IL-13_. Our results are in line with the presence of M2c, as well as a detected IL-10 secretion in the late state of the tissue repair phase^35,66,76^, which emphasized the importance of IL-10 in the myofibroblast dedifferentiation process, and hence, termination of the tissue repair phase.

**Fig. 7:**
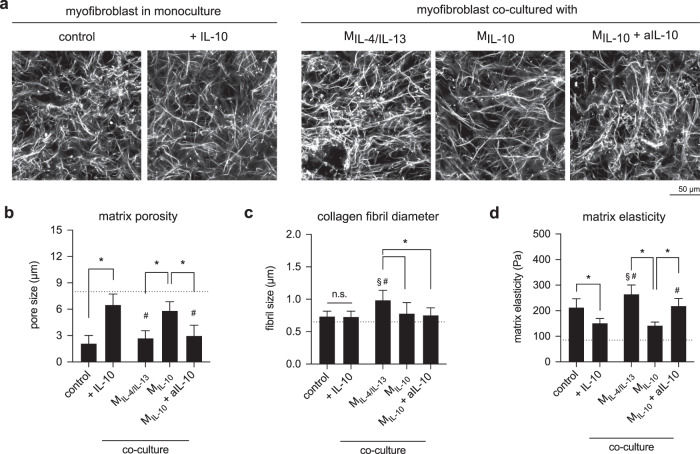
Matrix remodeling by myofibroblasts in the presence of IL-10 and co-culture with M2 subtypes. **a** Representative images of decellularized collagen matrices in mono and co-culture with M2 subtypes (scale bar: 50 μm). Decellularized collagen matrices were characterized regarding **b** matrix porosity characterized as mean pore size, **c** collagen fibril diameter, and **d** matrix elastic modulus. (Data are represented as mean ± SD; *significance level of *p* ≤ 0.05 using a one-way ANOVA followed by Tukey’s post hoc analysis.) The characters # and § represent the significance level of *p* ≤ 0.05 using a one-way ANOVA followed by Tukey’s post hoc analysis when compared to control, and treated samples with IL-10, respectively. For the quantification of matrix porosity and collagen fibril diameter, 10 different positions of each matrix condition were analyzed. Experiments were performed in four replicates.

The question arises whether de-differentiated myofibroblasts are able to remodel collagen matrices. To address this underlying question, we decellularized collagen matrices and analyzed their matrix porosity, fibril diameter, and matrix elasticity. Changes in matrix characteristics could be visually observed (Fig. 7a) and further quantitative analysis of matrix porosity showed that matrices of myofibroblasts treated with IL-10 and in co-culture with M_IL-10_ were significantly higher than those found in the co-culture with M_IL-4/IL-13_, and M_IL-10_ in the presence of aIL-10 (Fig. 7b). By analyzing collagen fibril diameter, we found that fibril diameter increased only in co-cultured conditions with M_IL-4/IL-13_, but not in monoculture and co-culture with M_IL-4/IL-13_ and M_IL-10_ with aIL-10 (Fig. 7b). This observation is similar to the one found in co-culture of fibroblast and M_IL-4/IL-13_ (Fig. 5), which might be caused by the enhanced pro-inflammatory milieu, tissue stiffness, microRNA^77,78^. Significantly lower matrix elasticity could be observed in myofibroblast treated with IL-10 and in co-culture with M_IL-10_ (Fig. 7d). Together with αSMA expression and matrix production, our matrix remodeling data suggest the dedifferentiation of myofibroblasts into fibroblast phenotypes in the presence of IL-10 and M_IL-10_. By blocking IL-10 using neutralizing antibody (aIL-10), we found that the expression of αSMA, matrix proteins, and the matrix remodeling remains at similar levels as myofibroblasts. It suggests that the dedifferentiation of myofibroblast is in response to the presence of supplemented or M_IL-10_ secreted IL-10. Our data emphasize the importance of IL-10 on the reduction of matrix remodeling by initiation of myofibroblast dedifferentiation.

### Fibroblast differentiation state is correlated with migratory behavior

**Fig. 8:**
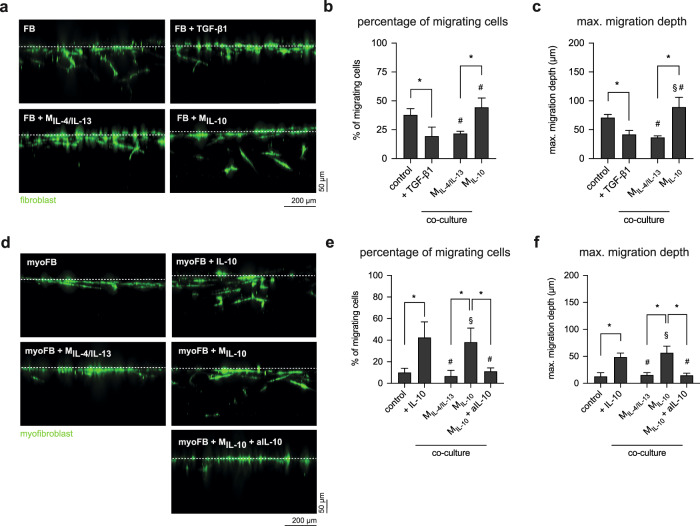
Migration of fibroblasts and myofibroblasts into 3D collagen matrices. Cells were stained with Phalloidin conjugated with Alexa Fluor-488 and DAPI, for visualization and quantification of cell migration into 3D collagen matrices, respectively. Representative images of *xz*-view of **a** fibroblast and **d** myofibroblast into 3D collagen matrices in mono- and co-culture with macrophages. Quantitative analysis of **b**, **e** percentage of migrating cells and **c**, **f** maximum migration depth of fibroblast and myofibroblasts. Cells found >20 μm beneath the matrix surface were counted as migrating cells. The maximum migration distance was defined as the distance crossed by 10% of all cells. Data are represented as mean ± SD; *significance level of *p* ≤ 0.05 using a one-way ANOVA followed by Tukey’s post hoc analysis. The characters # and § represent the significance level of *p* ≤ 0.05 using a one-way ANOVA followed by Tukey’s post hoc analysis when compared to control, and treated samples with TGF-β1 or IL-10, respectively. Three different positions of each matrix condition were analyzed. Experiments were performed in four replicates.

Besides matrix remodeling, another advantage of the 3D cell culture models is they allow to study cell migration. Migration of fibroblast cells during different stages of wound healing is important for tissue repair, but not much experimental evidence exists on the migratory behavior of fibroblasts at different differentiation stages and in co-culture conditions. We performed a migration analysis of both fibroblasts and myofibroblasts in mono- and co-culture conditions to address this underlying problem. Representative side-view images of fibroblast (Fig. 8a) and myofibroblast (Fig. 8d) show that fibroblast migration into 3D collagen matrices is significantly higher than that of myofibroblasts (TGF-β1-treated conditions), as required during early tissue repair phase. This data corroborate with our previous findings^8,66^. In addition, fibroblast co-culture with M_IL-10_ exhibited a similar migration capacity in both percentage of migrating cells (Fig. 8b) and maximal migration depth (Fig. 8c) when compared to untreated fibroblasts, but exhibited significantly higher migration when compared to myofibroblast and in co-culture with M_IL-4/IL-13_. In another experiment, modeling the late tissue repair phase, where pre-differentiated myofibroblasts were seeded onto 3D collagen matrices, we found that myofibroblasts showed limited migration into collagen matrices (Fig. 8d). However, an enhanced migration of myofibroblast in the presence of IL-10 and in co-culture with M_IL-10_ could be observed both in the migrating cell number (Fig. 8e) and maximal migration depth (Fig. 8f). Myofibroblasts co-culture with M_IL-4/IL-13_ and M_IL-10_ in the presence of aIL-10 showed similar migration patterns as those found in myofibroblast alone.

Overall, our data suggest that myofibroblast exhibits limited migration capacity when compared to fibroblast, which is well correlated with the high expression of αSMA and the contractile phenotypes, as previously published^8,17,79^. The dedifferentiation of myofibroblast into fibroblast phenotypes in the presence of IL-10 and in co-culture with M_IL-10_ could be confirmed by migratory behavior. In addition, by neutralizing IL-10 using antibody in the co-culture with M_IL-10_, the migratory behavior remains at the level of myofibroblasts, confirming the effect of IL-10 in myofibroblast dedifferentiation.

### General discussion and conclusion

**Fig. 9:**
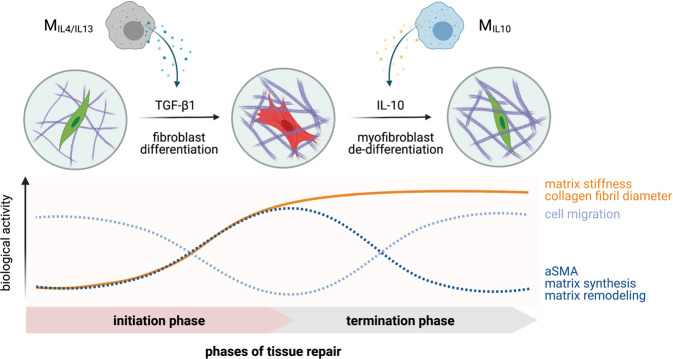
Schematic illustration of the proposed M2-associated functions in initiation and termination phases of tissue repair in 3D biomimetic wound-healing model. M_IL-4/IL-13_ macrophages control the initiation of tissue repair by modulating fibroblast differentiation via TGF-β1, while M_IL-10_ macrophages terminate the tissue repair by de-differentiating myofibroblasts back into fibroblasts via IL-10.

To establish wound-healing models for high-throughput assessment of pathobiological changes and testing of anti-fibrotic drugs, well-defined biosystems, including cells and their cell culture substrate, are required. Although primary human cells have become a major interest in the field of regeneration, they cannot be utilized to establish a well-defined wound model due to patient-to-patient variability as well as possible lack of data regarding their underlying activation stage. To overcome these issues, we used THP-1, a human monocytic cell line widely used as an established macrophages model in many biomedical studies such as wound repair, infection models, and immuno-oncology^80^. Although THP-1 based M1-like macrophage models have been extensively established, few studies attempted to study M2a- and M2c-like macrophages and address their cellular functions in the context of wound repair. In this work, we utilized collagen matrices as a biomimetic wound model and comprehensively analyzed and demonstrated that surface markers and cytokines secretion profile of M_IL-4/IL-13_ and M_IL-10_ is akin to M2a and M2c macrophages derived from human PBMC, respectively^22,28,52^. We showed that M_IL-4/IL-13_ can modulate tissue repair by controlled secretion of TGF-β1 to induce fibroblast differentiation, while M_IL-10_ macrophages secrete high amounts of IL-10 to resolve inflammation and tissue repair processes. Besides, we demonstrate that IL-10 can reverse myofibroblast into fibroblast phenotypes, as published elsewhere^35,66^. By neutralizing IL-10 with antibody in co-culture with M_IL-10_, no dedifferentiation of myofibroblast could be observed, emphasizing the role of IL-10 in resolution of the tissue repair phase. An illustration summary of our results in different tissue repair phases is depicted in Fig. 9. Interestingly, we found an increase in collagen fibril diameter only in the co-culture of myofibroblasts and M_IL-4/IL-13_ (M2a). This suggests that the contribution of M_IL-4/IL-13_ to the tissue repair process is through remodeling provisional wound tissue^22^. Our recent report demonstrates that thicker collagen fibrils could activate fibroblast differentiation via cell contractility^71^, advancing the development of tissue scarring and fibrosis. Alongside with that, a prolonged presence of M2a might also contribute to fibrosis^81,82^. Overall, our results pinpoint the importance of the co-culture model of fibroblast and macrophages for biomimetic wound healing, instead of fibroblast monoculture. In addition, our established biomimetic model can guide the development of well-defined high-throughput platforms for improving tissue healing and anti-fibrotic drugs testing, as well as other biomedical studies.

## Methods

### Preparation of cell-embedded 3D collagen matrix

To prepare collagen solution, Rat Tail type I Collagen (Advanced BioMatrix, Inc., CA, USA), 250 mM phosphate buffer (Merck KGaA, Munich, Germany), and 0.1% acetic acid (Merck KGaA, Germany) were mixed on ice at a concentration of 2 mg/ml, as previously published^72^. THP-1 monocytic cells (AddexBio, CA, USA) were deposited and resuspended in the prepared collagen solution and subsequently transferred onto a glutaraldehyde-coated coverslip (VWR, Darmstadt, Germany), allowing covalent binding of the collagen matrix via a lysine side chain^18^. The number of THP-1 cells used was 1 × 10^5^ cells per 3D collagen matrix. Collagen fibrillation was initiated immediately at 37 °C, 5% CO_2_ and 95% humidity for 45 min. Cell-embedded matrices were then rinsed three times with PBS (Merck KGaA, Germany).

### Cell culture and macrophages differentiation

Cell-embedded matrices were cultured in RPMI-1640 media supplemented with 10% fetal bovine serum (FBS), 1% sodium pyruvate, 0.01% of 2-mercaptoethanol, and 1% penicillin/streptomycin at 37 °C, 5% CO_2_, and 95% humidity. Cell culture media and supplements were purchased from Invitrogen, CA, USA.

THP-1 cells were differentiated into uncommitted macrophages using 300 nM phorbol 12-myristate 13-acetate (PMA; Merck KGaA, Germany)) in RPMI-1640 cell culture media without FBS supplement, according to the established protocol^18,40^. After 6 h, differentiation media was removed, cell-embedded matrices were washed with PBS, and rested for 24 h in RPMI-1640 without FBS supplement nor PMA. Afterwards, cells were activated for 48 h into two different macrophages subtypes M2_IL-4/IL-13_ by treatment with 20 ng/ml interleukin 4 (IL-4; Biolegend, CA, USA) and 20 ng/ml interleukin 13 (IL-13; Biolegend, USA), or into M2_IL-10_ by adding 20 ng/ml interleukin 10 (IL-10; Biolegend, USA).

After successful cell activation, cells were imaged in a bright-field mode by a DMi8 S microscope using ×20 long distance objective (NA 0.4; Leica, Germany). Cell area and cell aspect ratio (cell length/cell width) were analyzed using an automated image analysis toolbox^38^. At least 100 cells were analyzed. The experiment was performed at least in triplicates.

### Quantitative analysis of macrophage cell surface markers

To study the expression of cell surface markers, cells were harvested from 3D collagen matrices by digestion with 6 mg/ml type IV collagenase (Worthington, NJ, USA) for 10 min at the standard cell culture conditions. Prior cell staining, cells were incubated with Human TruStain FcX™-Fc Receptor Blocking Solution (5 µl of blocking solution in 100 µl of staining volume; Biolegend, USA) for 5 min at standard cell culture conditions. Cells were then stained with mouse anti-human antibodies against CD14, CD68, CD80, CD86, CD105, CD163, CD206, HLA-DR, and DRAQ7 (live/dead staining dye) for 30 min at standard cell culture conditions. Antibodies and DRAQ7 dye were diluted in cell culture medium at a ratio of 1:250. All antibodies used in this study were purchased from Biolegend, USA and listed with conjugated dye, clone, isotype, and catalog number in Supplementary Table 1. Stained cells were analyzed using Attune NxT Flow Cytometer equipped with an autosampler (Thermo Fisher Scientific, CA, USA). Compensation settings were applied prior to running the analysis. Data analysis was done using FlowJo software ((Becton, Dickinson and Company, NJ, USA). Gating strategy and representative histogram plot were depicted in Supplementary Fig. 2. gMFI of cell surface markers was evaluated. Experiments were performed in four replicates.

### Quantitative analysis of cytokine secretion profile

To analyze cytokines secreted by macrophages, cell culture supernatants were collected after cell activation. Bead-based multiplex immunoassay for IL-4, IL-2, CXCL10 (IP-10), IL-1β, TNF-α, MCP-1 (CCL-2), IL-17A, IL-6, IL-10, IFN-γ, IL-12p70, IL-8 (CXCL8), and TGF-β1 (Human essential immune response panel; Biolegend, USA) was utilized to quantify cytokines following instructions by the manufacturer. Samples were analyzed using an Attune NxT Flow Cytometer equipped with autosampler (Thermo Fisher Scientific, USA). Data analysis was done by applying a five-parameter curve fitting algorithm using LEGENDplex™ data analysis software (Biolegend, USA). Experiments were performed in six replicates.

### Co-culture with fibroblasts and myofibroblasts

For the co-culture with fibroblasts, macrophages activation media was removed, and matrices were washed three times with PBS prior to the introduction of fibroblasts/myofibroblasts. 1 × 10^4^ human primary dermal fibroblasts (ATCC, VA, USA) were seeded onto 3D collagen matrices with and without embedded macrophages and cultured for an additional 3 days at standard cell culture conditions.

For the co-culture with myofibroblasts, human primary dermal fibroblasts (ATCC, USA) were pre-differentiated into myofibroblasts by culturing onto 2D tissue culture plate in the presence of 10 ng/ml of recombinant human TGF-β1 (Biolegend, USA) for 3 days at standard cell culture conditions. Pre-differentiated myofibroblasts were quantified regarding their specific markers, namely αSMA, Coll1a1, and EDA-FN to ensure the differentiation (Supplementary Fig. 3). Afterwards, myofibroblasts were detached using TrypLE^TM^ Express Enzyme (1×) with phenol red. Similar to co-culture with fibroblasts, macrophages activation media was removed, and matrices were washed three times with PBS prior to the introduction of myofibroblasts. 1 × 10^4^ pre-differentiated myofibroblasts were seeded onto 3D collagen matrices with and without embedded macrophages. Co-culture of myofibroblasts and each macrophage subtype was performed for 3 days at standard cell culture conditions.

To verify the effect of IL-10 in myofibroblast dedifferentiation, we performed IL-10 blocking experiment in co-culture with M2_IL-10_, where 5 µg/ml of anti-human IL-10 antibody (aIL-10; Biolegend, USA) was supplemented to the cell culture media. Cells were culture for 3 days at standard cell culture condition prior to further analyses. All co-culture experiments were performed in four replicates.

### Cell staining and imaging

Cells were fixed with 4% paraformaldehyde (Biolegend, USA) for 10 min and permeabilized with 0.1% Triton X100 (Merck KGaA, Germany) for 10 min. Cells were washed three times with PBS after each step. Afterwards, cells were stained with Phalloidin conjugated with Alexa Fluor 594 (dilution 1:250 in PBS; Invitrogen, Carlsbad, CA, USA) and Hoechst-33342 (dilution 1:10,000 in PBS; Invitrogen, Carlsbad, CA, USA). For staining the αSMA, cells were blocked with 1% bovine serum albumin for 1 h at room temperature, incubated with mouse anti-human αSMA (dilution 1:250 in PBS; Biolegend, USA) overnight at 4 °C, and incubated with goat anti-mouse IgG conjugated with Alexa Fluor-488 (dilution 1:250 in PBS; Invitrogen, USA) for 2 h. Cells were washed three times with PBS after each step. Cell imaging was performed using an epi-fluorescence microscope (DMi8 S; Leica, Germany) using a ×20 long distance objective (NA 0.4; Leica, Germany).

### Gene expression analysis

Gene expression analysis was performed using an established protocol^18^. Briefly, RNA was extracted using TRIzol (Invitrogen, USA). The RNA concentration and the ratio of absorbance at 260 and 280 nm of cDNA were quantified using NanoDrop Spectrophotometers (Thermo Fisher Scientific, USA). Afterwards, RNA was converted into complementary DNA (cDNA) using high-capacity cDNA reverse transcription kit (Applied Biosystems, Foster City, CA, USA). The primers were synthesized from Bioneer (Republic of Korea). Quantitative polymerase chain reaction (qPCR) was performed using the SYBR Green PCR Master Mix (Applied Biosystems, USA) and was set as follows: denaturation for 5 min at 95 °C; 45 cycles of denaturation (95 °C, 15 s), annealing under primer-specific conditions (30 s), and target gene-specific extension (30 s at 72 °C). Fluorescence signals were measured for 20 s at 72 °C. *RPS26* gene was used as a reference gene. All primer sequences with accession number are listed in Supplementary Table 2. To confirm the specificity of the PCR products, melting curve analysis was performed at the end of each run. Both cDNA synthesis and qPCR were conducted using Stratagene Mx3005P (Agilent Technologies, CA, USA). The relative expression levels were calculated using the 2^−ΔΔCT^ method. Experiments were undertaken in four replicates.

### Topological and mechanical characterization of 3D collagen matrix

Cell-embedded matrices were decellularized by osmotic shock through incubation with distilled water for 1 h, as previously published^83^. Afterwards, cell-free collagen matrices were analyzed to assess their topological and mechanical properties. Briefly, for topological analysis, collagen matrices were stained with 50 µM of 5-(and-6)-carboxytetramethylrhodamine succinimidyl ester (TAMRA-SE, Sigma-Aldrich) and visualized by confocal laser scanning microscope (cLSM) (SP8; Leica, Germany) using ×40 water immersion objective (Leica, Germany), as published elsewhere^72^. The cLSM stacked images were analyzed using a custom-built MATLAB script (MATLAB 2019a; MathWorks, USA)^84^. For the quantification of pore size and fibril diameter of collagen matrices, stacked images of 10 different positions of each matrix condition were analyzed. Mechanical properties of cell-free matrices were analyzed using non-destructive rheological measurement using ElastoSens^TM^ Bio (Rheolution, Quebec, Canada)^18^. The topological and mechanical characterization were performed in four replicates.

### Cell migration into 3D collagen matrices

Fibroblast migration into 3D collagen matrices was quantitatively determined by analyzing Hoechst-33342 fluorescence signal from individual cell nuclei, as previously reported^72^. *Z*-stack images with an interval of 5 µm were gathered using an epi-fluorescence microscope with an automatic scanning stage (DMi8 S; Leica, Germany) using a ×10 dry objective (Leica, Germany). For each experiment, three different positions of each cell culture condition were analyzed. The *z*-position of cell nuclei as a function of migration distance was examined using a custom-built MATLAB script (MATLAB R2019b; MathWorks Inc., USA). Since macrophages exhibit relatively less actin expression when compared to fibroblasts/myofibroblasts, we use this phenomenon to distinguish fibroblasts for migration analysis. Cells located >20 µm below the collagen matrix surface were classified and counted as migrated cells. Maximum migration distance was defined as the distance that was crossed by 10% of all migrating cells. For visualization of cell migration into 3D collagen matrices in Fig. 8, fluorescence signal of Phalloidin conjugated with Alexa Fluor-488 was used. Experiments were performed in four independent replicates.

### Statistical analysis

Experiments were performed at least in four replicates unless otherwise stated. Error bars indicate standard deviation (SD). Levels of statistical significance were determined by a Mann–Whitney test or by a one-way ANOVA followed by Tukey’s post hoc analysis using GraphPad Prism 8 (GraphPad Software, USA). The significance level was set at *p* < 0.05.

### Reporting summary

Further information on research design is available in the Nature Research Reporting Summary linked to this article.

## Supplementary information

Supplementary Information

Reporting Summary

## Data Availability

The datasets generated during and/or analyzed during the current study are available from the corresponding author on reasonable request.

## Code availability

Custom script was built to measure (1) collagen microarchitecture and (2) maximum cellular migration depth into collagen hydrogels. Script 1 can be assessed here: https://git.sc.uni-leipzig.de/pe695hoje/topology-analysis and 2 can be made available upon reasonable request.
