# Supplementary material for: 3D in vitro M2 macrophage model to mimic modulation of tissue repair
Source: NPJ Regen Med. 2021 Nov 30;6:83. doi: 10.1038/s41536-021-00193-5 (PMC8633361; doi:10.1038/s41536-021-00193-5)
Supplement: Supplementary file 1 — Supplementary Information [file 41536_2021_193_MOESM1_ESM.pdf]

Supplementary Information

3D in vitro M2 macrophage model to mimic modulation of tissue repair

Jiranuwat Sapudom[1], Shaza Karaman[1,2], Walaa KE Mohamed[1], Anna Garcia-Sabaté[1],
Brian C Quartey[1], Jeremy CM Teo[1,3,*]

[1] Laboratory for Immuno Bioengineering Research and Applications, Division of Engineering, New York University Abu Dhabi, Abu Dhabi, UAE.

[2] Department of Biomedical Engineering, Imperial College London, UK.

[3] Department of Mechanical and Biomedical Engineering, Tandon School of Engineering, New York University USA.

* Corresponding author

Keywords:

Macrophage, fibroblast differentiation, myofibroblast dedifferentiation, tissue repair, collagen matrix

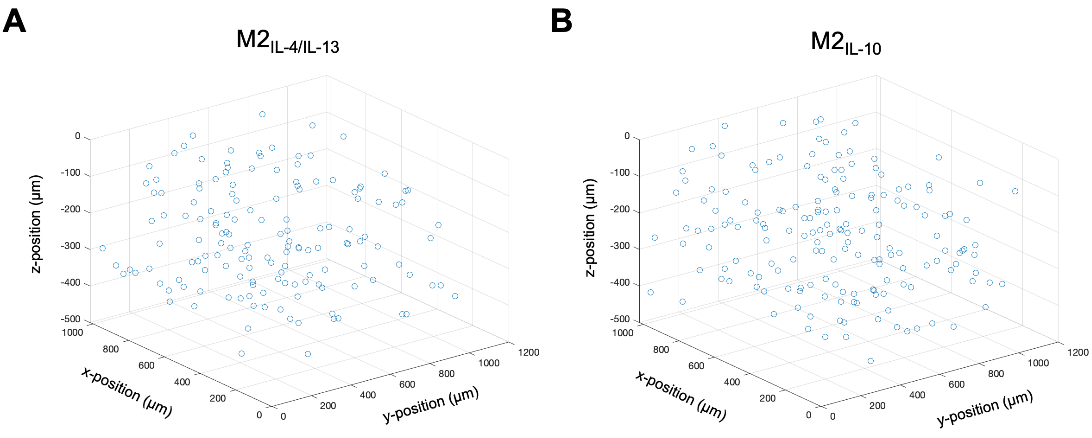

Supplementary Figure 1: Representative analysis of distribution of **(A)** M2$_{IL-4/IL-13}$ AND **(B)** M2$_{IL-10}$ within 3D collagen matrices after 3 days of activation. The analysis was performed using DAPI fluorescence signal using custom-built image analysis toolbox. Each circle represents single cells.

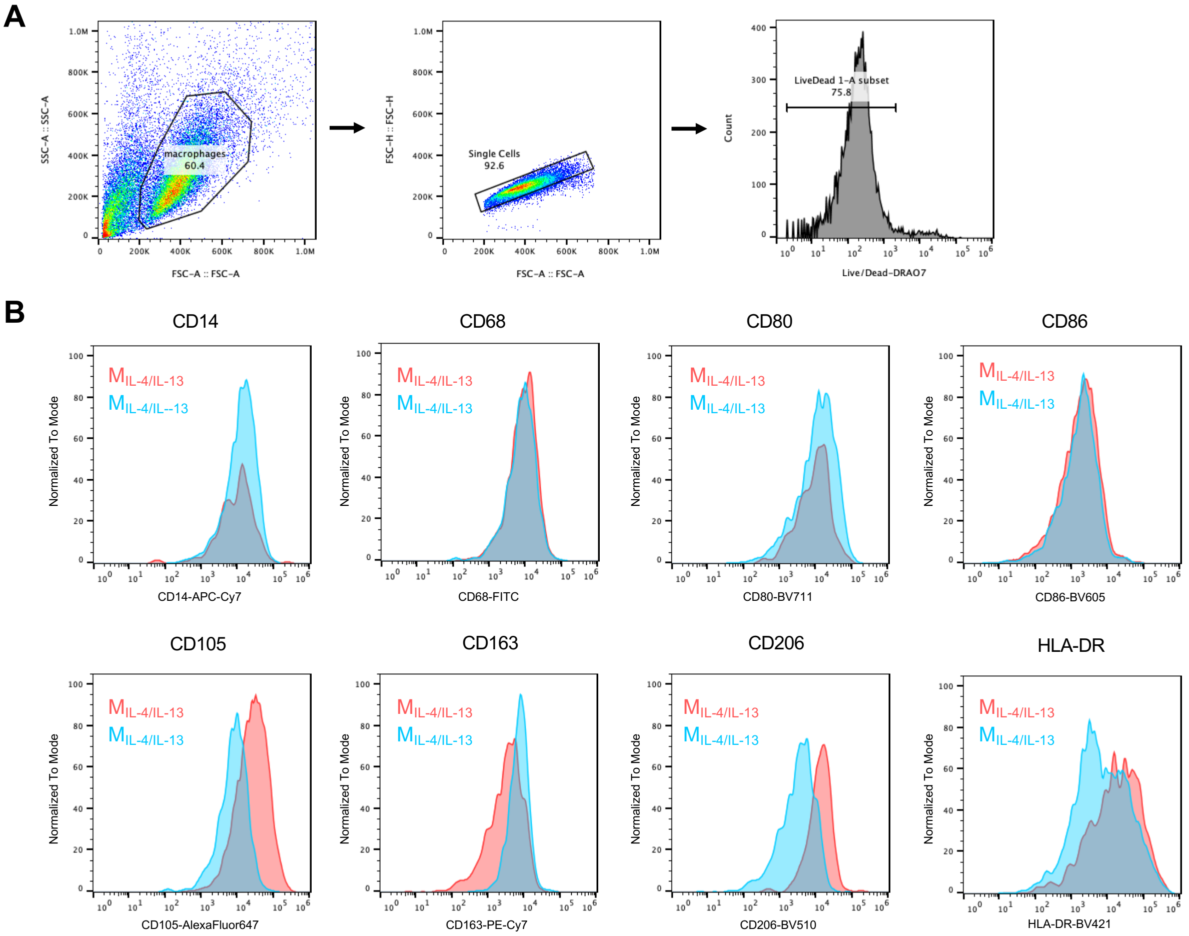

Supplementary Figure 2: Representative gating strategy and histogram plots of cell surface markers. **(A)** Gating strategy. Initial cell populations were gated for a live population using FSC and SSC plot of cell only sample. The gate was set to remove cell debris and dead cells (small FSC v SSC) and large clumps or aggregates of cells (large FSC or SSC) and used across all samples. Gate was cross-checked against DRAQ-7 cell viability-stained sample. This live population was then used in fluorescent histograms. **(B)** Representative histogram plots of cell surface markers used in this study. The visualization was performed using FlowJo Software (BD, USA).

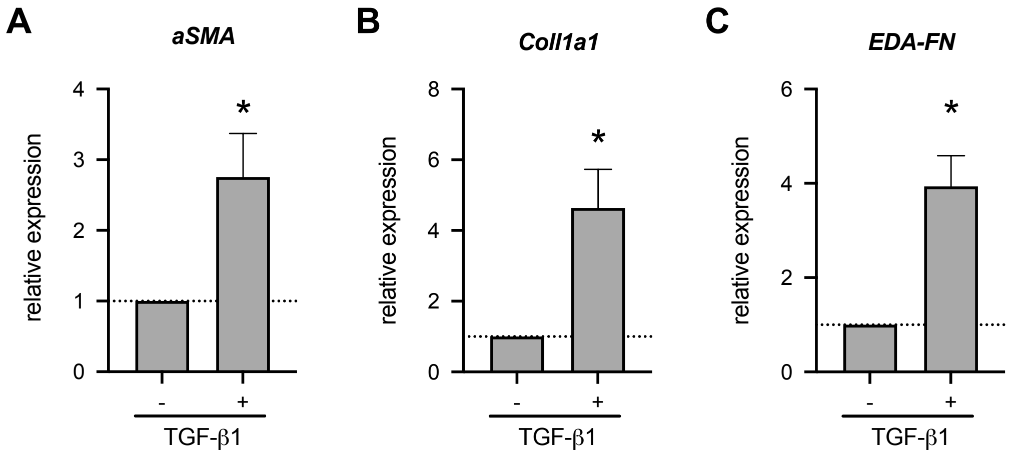

Supplementary Figure 3: Quantitative analysis of pre-differentiated myofibroblasts on tissue culture plastic. Gene expression analysis of (C) *αSMA*, (D) *Coll1a1* and (E) *EDA-FN* were performed using qPCR. Data are represented as mean ± SD; * – significance level of $p < 0.05$ using a one-way ANOVA followed by Tukey's post hoc analysis compared to untreated sample. Experiments were performed in triplicates.

Supplementary Table 1: Antibodies used in the study. All antibodies are purchased from Biolegend.

Marker	Color / Format	Host / Target	Isotype	Clone	Catalog Nr.
CD14	APC-Cy7	Mouse anti-Human	IgG1 κ	63D3	367108
CD68	FITC	Mouse anti-Human	IgG2b κ	Y1/82A	333805
CD80	Brilliant Violet 711	Mouse anti-Human	IgG1 κ	2D10	305236
CD86	Brilliant Violet 605	Mouse anti-Human	IgG1 κ	BU63	374214
CD105	Alexa Fluor 647	Mouse anti-Human	IgG1 κ	43A3	323212
CD163	PE-Cy7	Mouse anti-Human	IgG1 κ	GHI/61	333614
CD206	Brilliant Violet 510	Mouse anti-Human	IgG1 κ	15-2	321138
HLA-DR	Brilliant Violet 421	Mouse anti-Human	IgG2a κ	L243	307636

Supplementary Table 2: RT-qPCR primer sequence.

**Genes**	**Forward primer Sequence (5′ → 3′)**	**Reverse primer Sequence (5′ → 3′)**	**Accession number**
RPS26	CAATGGTCGTGCCAAAAAG	TTCACATACAGCTTGGGAAGC	NM_001029
αSMA (ACTA2)	AGACCCTGTTCCAGCCATC	TGCTAGGGCCGTGATCTC	NM_001141945.1
Coll1a1	GTCGCACTGGTGATGCTG	GGTGGTGTCCACCTCGAG	NM_002421.3
EDA-FN	CCAGTCCACAGCTATTCCTG	AAACTGCTGCAAGAGGTTATTCCT	NM_002026.2
